# Differential Time-of-Day Effects of Caffeine Capsule and Mouth Rinse on Cognitive Performance in Adolescent Male Volleyball Athletes: A Randomized Crossover Investigation

**DOI:** 10.3390/life16010033

**Published:** 2025-12-25

**Authors:** Salma Belhaj Amor, Wissem Dhahbi, Houda Bougrine, Manel Bessifi, Vlad Adrian Geantă, Vasile Emil Ursu, Khaled Trabelsi, Nizar Souissi

**Affiliations:** 1 Physical Activity, Sport and Health Research Unit (UR18JS01), National Observatory of Sports, Tunis 1003, Tunisia; salmabhamor@gmail.com (S.B.A.); houdabougrine@live.fr (H.B.); n_souissi@yahoo.fr (N.S.); 2 High Institute of Sports and Physical Education of El Kef, University of Jendouba, El Kef 7100, Tunisia; 3 Research Unit (UR22JS01) “Sport Sciences, Health and Movement”, High Institute of Sport and Physical Education of Kef, University of Jendouba, El Kef 7100, Tunisia; wissem.dhahbi@gmail.com (W.D.); manelbessifii@gmail.com (M.B.); 4 Training Department, Police College, Qatar Police Academy, Doha 7157, Qatar; 5 High Institute of Sport and Physical Education of Gafsa, Gafsa University, Gafsa 2112, Tunisia; 6 Department of Physical Education and Sport, Faculty of Physical Education and Sport, Aurel Vlaicu University of Arad, 310025 Arad, Romania; 7 Doctoral School of Sport Science and Physical Education, Pitești University Center, National University of Science and Technology Politehnica Bucharest, 110253 Pitești, Romania; 8 Department of Physical Education and Sport, Faculty of Law and Social Sciences, University “1 Decembrie 1918” of Alba Iulia, 510009 Alba Iulia, Romania; 9 Research Laboratory Education, Motricité, Sport et Santé, EM2S, LR19JS01, High Institute of Sport and Physical Education of Sfax, University of Sfax, Sfax 3000, Tunisia; trabelsikhaled@gmail.com; 10 Department of Movement Sciences and Sports Training, School of Sport Science, The University of Jordan, Amman 28002, Jordan; 11 High Institute of Sport and Physical Education, University of Manouba, Ksar-Said, Mannouba 2010, Tunisia

**Keywords:** methylxanthines, orosensory stimulation, psychomotor speed, ergogenic aids, diurnal variation, youth sports

## Abstract

Caffeine is widely used to enhance cognitive performance, but its efficacy may vary with the administration route and circadian timing. This study compared the acute effects of caffeine capsule ingestion and caffeine mouth rinsing on cognitive performance across morning, midday, and evening sessions in well-trained, adolescent male volleyball players. Twenty-four athletes completed three randomized, double-blind, crossover trials involving a caffeine capsule (3 mg·kg^−1^), a caffeine mouth rinse of the same dose (expectorated), and a placebo. Cognitive performance was assessed using simple and choice reaction time tests and the Stroop task, alongside a side-effects questionnaire. Both caffeine forms improved performance versus the placebo, with the greatest enhancements occurring at midday and moderate benefits evident in the morning. Capsule ingestion produced the most consistent improvements across reaction speed and executive control, whereas mouth rinsing elicited smaller, task-dependent effects, particularly at midday. No consistent or practically relevant benefits were observed for either caffeine condition in the evening, when cognitive performance was naturally highest. Side effects were mild and infrequent, with occasional headaches after capsule ingestion. These findings indicate that caffeine capsules most effectively enhance cognitive performance when baseline alertness is suboptimal, while caffeine mouth rinsing represents a practical ingestion-free alternative with moderate efficacy.

## 1. Introduction

Caffeine is among the most widely used ergogenic aids in sport, with convergent evidence showing small-to-moderate acute performance benefits at doses of ~3–6 mg·kg^−1^ when appropriately timed (typically ~60 min pre-task) [1]. It can be consumed in various forms, including capsules, beverages, gels, gums, powders, and mouth rinses, each differing in absorption kinetics, onset of action, and practical applicability [2]. Beyond physical performance, caffeine enhances attention and vigilance and may accelerate reaction speed in athletic contexts, although effects vary with task demands and experimental conditions [3]. These effects are primarily mediated through adenosine receptor antagonism, which increases arousal and psychomotor readiness [4].

Cognitive and motor performance vary across the day. Circadian rhythmicity and sleep–wake homeostasis jointly shape alertness and executive function, producing slower psychomotor responses and weaker cognitive control in the morning (sleep inertia, lower core temperature) with recovery toward afternoon/evening plateaus [5]. In sport, diurnal trends show early-day nadirs and late-afternoon peaks, modulated by chronotype [6].

Previous studies indicated that caffeine ingestion can partially offset morning troughs in both cognitive and high-intensity exercise outcomes, narrowing time-of-day (TOD) differences [7]. Accordingly, Bougrine and colleagues reported that caffeine’s ergogenic effects could be greater in the morning than in the evening and that dose moderated both efficacy and side-effects in athletic populations [8,9]. These issues are particularly relevant to volleyball, which imposes acute perceptual-cognitive demands. Indeed, athletes must rapidly detect and track ball flight, anticipate opponents’ intentions, select responses, and inhibit premature actions under tight temporal constraints. Better basic cognitive functioning (e.g., faster simple reactions, stronger interference control) is associated with superior sport-specific performance in youth volleyball, underscoring the applied value of interventions that acutely support these capacities [10,11].

Caffeine mouth rinsing may preserve ergogenic potential by activating orosensory pathways linked to central arousal and reward, while minimizing gastrointestinal or sleep-related side effects [12]. However, evidence for its ergogenic effects remains inconsistent. Systematic reviews report protocol-dependent outcomes, with benefits varying according to rinse duration, nutritional state, and task characteristics [13,14,15]. Most trials show null or trivial effects versus placebo [16,17], leaving mechanistic and practical uncertainties.

Additionally, the available evidence was drawn almost exclusively from adult populations [14], leaving limited insight into whether these strategies differ in efficacy or practicality for younger athletes. Adolescents may demonstrate distinct sensitivity to caffeine due to ongoing neurodevelopment of prefrontal and reward circuitry [18], different sleep–wake patterns and greater susceptibility to circadian misalignment [19,20], and potentially increased vulnerability to caffeine-related side effects, such as sleep disruption and mood disturbances [21]. These characteristics highlight the relevance of studying non-ingestive caffeine modalities, including mouth rinsing, in adolescent athletes.

Therefore, this study aimed to compare the acute effects of isodosed (3 mg·kg^−1^) caffeine capsules, caffeine mouth rinse, and placebo on reaction speed and executive control at 08:00, 12:00, and 18:00 in trained adolescent male volleyball players. Based on mechanistic and chronobiological considerations, we hypothesized that (i) capsule ingestion would elicit larger benefits than mouth rinsing across cognitive outcomes, and (ii) benefits would be greater in the morning and at midday (when baseline alertness and executive control are lower) than in the evening, thereby attenuating diurnal performance gaps [1,5,9,22,23,24].

## 2. Materials and Methods

### 2.1. Participants

An a priori power analysis using G*Power software (version 3.1.9.7) (repeated-measures ANOVA, within-subjects design; 3 levels for condition × 3 levels for time of day) determined the required sample size. The expected effect size (f = 0.25) derived from adult athlete caffeine-cognition studies [7,25], with extrapolation to adolescents justified by (1) preserved adenosine receptor sensitivity despite developmental differences [4], (2) lower habitual caffeine exposure in youth (reducing tolerance), and (3) recent trials demonstrating comparable effect magnitudes in adolescent athletes [8,9]. The 3 mg·kg^−1^ dose was chosen to balance ergogenic efficacy against adolescent-specific safety considerations and in accordance with the methodology outlined by Cooper et al. [26]. Furthermore, although 6 mg·kg^−1^ doses maximize performance effects in adults [1], our participants’ developmental status (age 16–18 years) and low habitual caffeine consumption (1.14 mg·kg^−1^·day^−1^) warranted conservative dosing. Recent trials in youth athletes demonstrated that 3 mg·kg^−1^ elicited moderate cognitive improvements with minimal side effects, whereas higher doses increased adverse events without proportional benefits in low-consumer populations [8,25]. With α = 0.05, statistical power (1−β) = 0.80, and an assumed within-subject correlation of r = 0.50, the required sample size was N = 21 [27]. To account for potential dropouts, 24 athletes were recruited. All randomized participants completed the protocol and were included in the analyses.

Twenty-four well-trained male volleyball players (age: 16.9 ± 0.7 years, range 16.0–18.0 years) volunteered. Their mean stature, body mass, and body mass index (BMI) were 177.0 ± 3.4 cm (range 171.0–184.0 cm), 71.0 ± 4.2 kg (range 64.0–80.0 kg), and 22.7 ± 1.7 kg·m^−2^ (range 19.8–26.1 kg·m^−2^), respectively. Participants had an average volleyball training experience of 7.0 ± 1.0 years (range 5.0–9.0 years). Inclusion criteria were as follows: ≥5 years of competitive volleyball experience, participation in ≥4 training sessions·week^−1^ during the preceding 6 months, habitual caffeine intake <3 mg·kg^−1^·day^−1^, good sleep quality (PSQI ≤ 5 [28], and “neither-type” chronotype on the Horne-Östberg Morningness-Eveningness Questionnaire (MEQ) [29]. Exclusion criteria were the following: current smoking or alcohol use, chronic disease or regular medication, injury within the last 3 months, stimulant or narcotic use, restrictive dieting, known caffeine allergy, or extreme chronotype. Habitual caffeine intake (4-week semi-quantitative questionnaire [30]) averaged 1.14 ± 0.11 mg·kg^−1^·day^−1^, classifying participants as mild caffeine users [31]. All participants (and parents or guardians for minors) provided written informed consent. The protocol was approved by the University of Jendouba Ethics Committee (approval number: C-0018/2024, approval date: 18 November 2024) and conformed to the Declaration of Helsinki and its subsequent amendments.

### 2.2. Experimental Design

We used a randomized, double-blind, placebo-controlled, crossover design to reduce interindividual variability and control for order and learning effects. The three conditions (caffeine capsule [CAFcap], caffeine mouth rinse [CAFrinse], and placebo [PLA]) were completed in a counterbalanced Latin-square order generated with an online randomization tool [32], with allocation concealed by an investigator independent of data collection and codes revealed only after analyses. Before testing, participants attended a familiarization session under identical environmental conditions during which procedures were rehearsed, anthropometrics were recorded to individualize caffeine dose, and questionnaires were administered (habitual caffeine intake; Pittsburgh Sleep Quality Index, PSQI; Morningness-Eveningness Questionnaire, MEQ). Only athletes with good sleep quality (PSQI ≤ 5) were retained [28]; chronotype was documented with the MEQ [29]. Pre-trial controls required maintaining habitual sleep–wake schedules for ≥48 h, abstaining from vigorous exercise, alcohol, and caffeine for 24 h, and consuming the last meal ≥3 h before arrival. To limit sleep inertia, 08:00 sessions were scheduled ≥90 min after each participant’s habitual wake time [24,33].

On each test day, interventions were prepared and coded by an independent investigator. CAFcap (3 mg·kg^−1^ caffeine anhydrous; Biotech USA, Budapest, Hungary) was ingested with 250 mL water, followed by 30 min absorption (plasma Tmax ~45 min [1,34]). CAFrinse consisted of an isodosed caffeine solution (3 mg·kg^−1^ in 250 mL of water) swilled for 10–15 s and then completely expectorated. Testing commenced immediately afterward to capture potential orosensory effects described in the mouth-rinse literature [14,35,36]. PLA matched the caffeine conditions in appearance, taste, and volume. It consisted of a cellulose capsule identical in shape and color to the caffeine capsule and a noncaffeinated rinse prepared with plain water containing a small amount of quinine and food-grade flavoring to mimic the characteristic bitterness of caffeine. Sessions were separated by ≥72 h to preclude carryover, consistent with an adult caffeine half-life of ~3–7 h [37,38]. Testing occurred on nonconsecutive days at 08:00, 12:00, and 18:00, with time-of-day order counterbalanced. These a priori points were chosen to sample diurnal variation in alertness, core temperature, and executive performance [39,40]. Upon arrival, participants rested seated for 10 min in a quiet thermoneutral room (~22 °C, ~47% relative humidity). Immediately before the cognitive battery (after the 30 min absorption for CAFcap; timing matched for CAFrinse and PLA), oral temperature (OT) was measured sublingually using a calibrated digital clinical thermometer (Omron Healthcare Co., Ltd., Kyoto, Japan; manufacturer-stated accuracy ±0.05 °C) with the probe kept under the tongue for at least 3 min. Cognitive testing followed a fixed sequence with standardized recovery periods: simple reaction time test, two-minute seated rest, choice reaction time test, three-minute seated rest, and the Stroop test battery (neutral condition). Participants completed a standardized side-effects checklist including items such as jitteriness, gastrointestinal discomfort, and palpitations.

**Figure 1:**
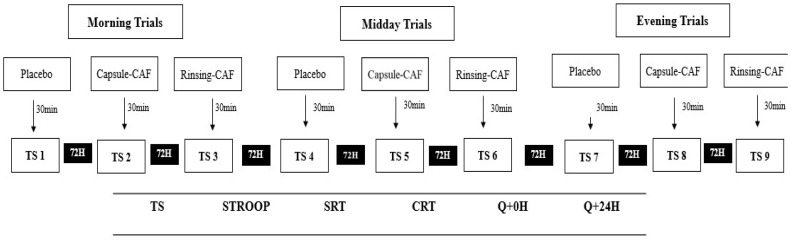
Randomized, double-blind, placebo-controlled crossover design depicting counterbalanced administration of supplementation conditions across three times of day and fixed cognitive test sequence with side-effects assessment protocol.

Figure 1 depicts the randomized, double-blind, placebo-controlled crossover schedule, showing the counterbalanced administration of placebo, caffeine capsule (3 mg·kg^−1^), and caffeine mouth rinse across morning (08:00), midday (12:00), and evening (18:00) sessions (TS1–TS9), together with the fixed test order (SRT, CRT, Stroop) and side-effects questionnaires at 0 h and 24 h.

### 2.3. Supplementation Protocols

On each test day, participants received one of three interventions in a randomized, double-blind, placebo-controlled, crossover manner: (i) caffeine capsule (CAFcap) containing caffeine anhydrous at 3 mg·kg^−1^ body mass, swallowed with 250 mL of water and followed by 30 min rest before testing. Capsules were supplied by BiotechUSA Kft. (Budapest, Hungary). (ii) Caffeine mouth rinse (CAFrinse) consisting of an aqueous solution containing 3 mg·kg^−1^ of caffeine dissolved in 250 mL of water, swilled for 10–15 s and then completely expectorated (no ingestion). Testing commenced immediately afterward to assess the orosensory effects described in previous mouth-rinse research [14,15,16]. (iii) Placebo (PLA) comprising an identical capsule and rinse without caffeine, matched for appearance, taste, volume, and color to ensure blinding.

All interventions were prepared and coded by an independent investigator not involved in data collection. Sessions were separated by ≥72 h to prevent carryover effects, consistent with caffeine’s adult half-life (~3–7 h) [37]. Participants were instructed to abstain from caffeine, alcohol, and vigorous exercise for 24 h before each trial and to maintain their habitual sleep–wake schedule for 48 h. Compliance was verified verbally at −48 h and −24 h and confirmed upon arrival. Immediately after completing the cognitive battery, participants completed a nine-item yes/no side-effects questionnaire [41] repeated 24 h later.

### 2.4. Cognitive Outcomes

Cognitive performance was assessed with three tasks administered on a standardized computerized platform (INRP freeware, v4.05; Tilquin): simple reaction time (SRT), choice reaction time (CRT), and Stroop test [42]. The SRT indexed basic psychomotor speed and alertness with 40 trials and a variable intertrial interval of 750–1350 ms; the primary outcome was mean latency for correct responses. The CRT assessed decision speed under perceptual load by requiring rapid identification of a target amid distractors with millisecond-level timing. The Stroop test comprised three consecutive 45 s subtasks (neutral reading, congruent color naming, and incongruent color–word interference), yielding interference time and error counts as main outcomes. These tasks are widely used and sensitive in sport contexts. SRT and CRT validly capture processing speed and decision-making under time pressure [43]; in volleyball, perceptual-cognitive abilities such as visual scanning, rapid reaction, and decision-making are central, with light-based perception-action training studies showing meaningful reaction-time improvements that support the ecological validity of reaction-based tasks [44]. The Stroop remains a robust index of selective attention, cognitive flexibility, and inhibitory control, with recent app-based tools (e.g., EncephalApp Stroop) showing high validity and reliability in physically active populations [45].

### 2.5. Statistical Analysis

For each continuous outcome (simple reaction time [SRT], choice reaction time [CRT], Stroop interference time, and Stroop interference errors), we performed a two-way repeated-measures ANOVA with within-subjects factors Condition (PLA, CAFcap, CAFrinse) and Time-of-day (08:00, 12:00, 18:00). Model assumptions were examined on residuals (Shapiro–Wilk for normality) and with Mauchly’s test for sphericity; Greenhouse–Geisser corrections were applied when sphericity was violated. When a main effect or interaction was significant, planned pairwise comparisons with Bonferroni adjustment were conducted at each time point or within each condition, as appropriate. Statistical significance was set at *p* ≤ 0.05 (two-tailed). Effect sizes are reported as partial η^2^p for omnibus tests (small ≈ 0.01, medium ≈ 0.06, large ≥ 0.14) [46] and as dz for all within-subjects pairwise contrasts, computed as mean difference divided by the standard deviation of paired differences, with benchmarks small ≈ 0.20, medium ≈ 0.50, large ≥ 0.80 [47]. Descriptive data are reported as mean ± SD. Adverse events (yes/no) were analyzed within each time of day using Cochran’s Q across the three paired conditions. When the global test was significant, pairwise McNemar tests (with continuity correction and exact *p* values when discordant counts were small) were performed with Holm adjustment for multiple comparisons. Results are reported as *n* (%) with the numbers of discordant pairs (b01/b10). Carryover effects were evaluated by comparing first-session performance across conditions using one-way ANOVA. No significant condition effects emerged for SRT (F(2, 21) = 0.43, *p* = 0.656), CRT (F(2, 21) = 0.68, *p* = 0.517), Stroop interference time (F(2, 21) = 1.12, *p* = 0.343), or Stroop errors (F(2, 21) = 0.89, *p* = 0.425), confirming that the ≥72 h washout interval (exceeding caffeine’s 3–7 h half-life by tenfold [37,38]) prevented residual effects. Statistical significance was set at 0.05 for two-tailed tests. All analyses were performed using STATISTICA software, version 13.0 (StatSoft, Maisons-Alfort, France).

## 3. Results

### 3.1. Oral Temperature

A significant main effect of time of day was observed for oral temperature (F(2, 46) = 20.90, *p* < 0.0001, ηp^2^ = 0.476), whereas no significant effect of condition (F(2, 46) = 2.07, *p* = 0.138, ηp^2^ = 0.083) and no time-by-condition interaction (F(4, 92) = 0.43, *p* = 0.784, ηp^2^ = 0.018) were found. Post hoc analyses revealed that at 12:00, oral temperature was significantly higher than at 08:00 (Δ = +0.31 ± 0.30 °C; q = 8.40; *p* < 0.001; dz = 1.04) and also higher than at 18:00 (Δ = +0.27 ± 0.23 °C; q = 7.32; *p* < 0.001; dz = 1.16). However, there was no difference between 18:00 and 08:00 (Δ = +0.04 ± 0.24 °C; q = 1.08; *p* = 0.709; dz = 0.17). Comparisons between conditions at each time point were nonsignificant (all *p* ≥ 0.57), confirming that the supplementation protocols did not influence oral temperature.

### 3.2. Simple Reaction Time (SRT)

A significant main effect of time of day was found for simple reaction time (F(2, 48) = 8.27, *p* = 0.0008, ηp^2^ = 0.256), along with a significant main effect of condition (F(2, 48) = 4.33, *p* = 0.0187, ηp^2^ = 0.153) and a significant time-by-condition interaction (F(4, 96) = 3.79, *p* = 0.0066, ηp^2^ = 0.136). Post hoc analyses revealed that at 08:00, reaction times were significantly faster in the caffeine capsule condition compared with both placebo (−11.08 ± 17.34 ms, dz = 0.64, *p* = 0.008) and caffeine rinse (−9.10 ± 13.82 ms, dz = 0.66, *p* = 0.009). At 12:00, the caffeine capsule again produced faster responses than placebo (−10.47 ± 17.84 ms, dz = 0.59, *p* = 0.020) and caffeine rinse (−10.56 ± 21.60 ms, dz = 0.49, *p* = 0.042). No between-condition differences were observed at 18:00 (*p* > 0.05). Within-condition analyses showed that under placebo, reaction times were shorter at 18:00 compared with 08:00 (−17.87 ± 27.45 ms, dz = 0.65, *p* = 0.007) and 12:00 (−14.47 ± 19.26 ms, dz = 0.75, *p* = 0.003). Under the caffeine capsule condition, no significant differences were found across times of day, whereas under the caffeine rinse condition, reaction times were faster at 18:00 compared with 08:00 (−17.77 ± 25.42 ms, dz = 0.70, *p* = 0.004) and 12:00 (−16.43 ± 21.26 ms, dz = 0.77, *p* = 0.002).

Table 1 and Table 2 presents values (mean ± SD) of oral temperature, simple reaction time (SRT), choice reaction time (CRT), and Stroop test scores registered during the three times of day (08:00, 12:00, and 18:00) throughout three testing conditions: placebo, capsule-caffeine intake, and mouth-rinsing caffeine intake.

**Figure 2:**
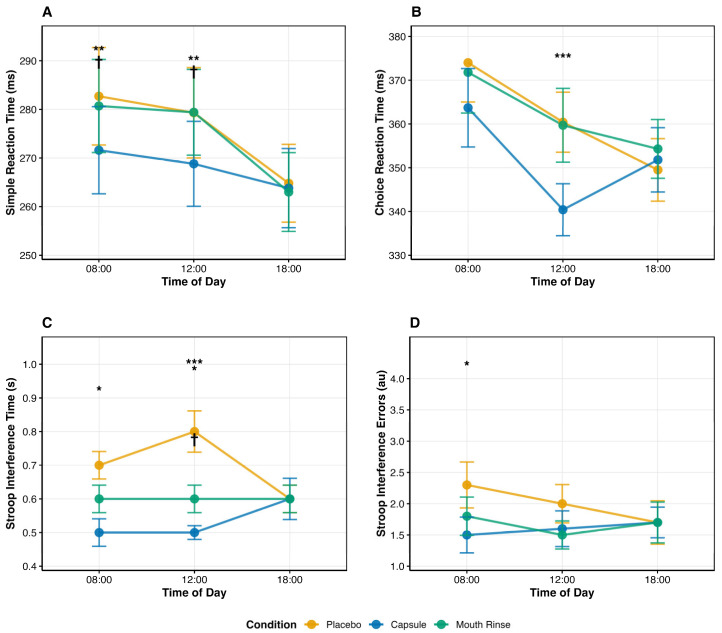
Time-of-day × condition interaction effects on cognitive performance outcomes. Error bars represent ±1 SEM (*n* = 24). Asterisks denote significant pairwise differences versus placebo at the same timepoint: * *p* < 0.05, ** *p* < 0.01, *** *p* < 0.001. Daggers denote significant differences versus caffeine mouth rinse at the same timepoint: † *p* < 0.05. (**A**) Simple reaction time (SRT) showed significant capsule benefits at 08:00 and 12:00 versus both placebo and mouth rinse. (**B**) Choice reaction time (CRT) exhibited capsule superiority only at 12:00 versus placebo. (**C**) Stroop interference time improved with capsules at 08:00 and 12:00, with mouth rinse showing modest midday benefits. (**D**) Stroop interference errors decreased with capsules at 08:00 only. No between-condition differences emerged at 18:00 for any outcome, indicating performance ceiling effects during the circadian optimum.

Figure 2 illustrates these condition × time-of-day interactions across all cognitive outcomes, revealing consistent midday benefits for capsules and minimal evening effects across conditions.

### 3.3. Choice Reaction Time (CRT)

Significant main effects of time of day (F(2, 48) = 4.31, *p* = 0.0189, ηp^2^ = 0.152) and condition (F(2, 48) = 5.66, *p* = 0.0062, ηp^2^ = 0.191) were observed for choice reaction time, together with a significant time-by-condition interaction (F(4, 96) = 3.33, *p* = 0.0135, ηp^2^ = 0.122). Post hoc analyses indicated that at 12:00, responses were significantly faster in the caffeine capsule condition compared with placebo (−19.98 ± 28.38 ms, dz = 0.70, *p* = 0.0057), whereas no between-condition differences were found at 08:00 or 18:00 (*p* > 0.05). Within-condition analyses revealed that under placebo, reaction times were shorter at 18:00 compared with 08:00 (−24.55 ± 31.87 ms, dz = 0.77, *p* = 0.002), and under the caffeine capsule condition, responses were faster at 12:00 than at 08:00 (−23.33 ± 35.29 ms, dz = 0.66, *p* = 0.009). No other within-condition contrasts reached significance.

### 3.4. Stroop Interference Time

No significant main effect of time of day was found for Stroop interference time (F(2, 46) = 0.07, *p* = 0.931, ηp^2^ = 0.003). However, a significant main effect of condition (F(2, 46) = 8.21, *p* = 0.0009, ηp^2^ = 0.263) and a significant time-by-condition interaction (F(4, 92) = 3.14, *p* = 0.018, ηp^2^ = 0.120) were observed. Post hoc analyses revealed that at 08:00, performance was faster in the caffeine capsule condition compared with placebo (−0.156 ± 0.237 s, dz = 0.66, *p* = 0.011). At 12:00, both the caffeine capsule (−0.224 ± 0.279 s, dz = 0.80, *p* = 0.002) and caffeine rinse (−0.165 ± 0.338 s, dz = 0.49, *p* = 0.049) resulted in shorter interference times than placebo, with the capsule also outperforming the rinse (+0.059 ± 0.139 s, dz = 0.42, *p* = 0.0487). No between-condition differences were detected at 18:00 (*p* > 0.05), and within-condition analyses revealed no significant effects of time of day.

### 3.5. Stroop (Interference)—Errors

A significant main effect of condition was observed for Stroop interference errors (F(2, 46) = 4.98, *p* = 0.011, ηp^2^ = 0.178), whereas no significant main effect of time of day (F(2, 46) = 0.17, *p* = 0.846, ηp^2^ = 0.007) and no time-by-condition interaction (F(4, 92) = 1.16, *p* = 0.333, ηp^2^ = 0.048) were found. Post hoc analyses indicated that at 08:00, the caffeine capsule condition resulted in fewer errors compared with placebo (−0.71 ± 1.20 errors, dz = 0.59, *p* = 0.024), whereas no significant differences were observed among conditions at 12:00 or 18:00.

### 3.6. Side Effect

Cochran’s Q identified significant condition effects for headache (χ^2^(2) = 6.50, *p* = 0.039) and perceived performance improvement (χ^2^(2) = 8.24, *p* = 0.016) at 12:00 only. Post hoc tests showed CAFcap increased headache incidence versus placebo (25.0% vs. 0%, *p* = 0.031) and perceived improvement versus placebo (50.0% vs. 12.5%, *p* = 0.012) at midday. No participants reported sleep disturbances at 24 h follow-up, including after 18:00 sessions. All other symptoms (tachycardia, anxiety, gastrointestinal issues) remained below 8.3% across conditions and times.

Table 3 presents adverse events by time of day and condition (N = 24). Values are given as a percentage of participants.

## 4. Discussion

This randomized, double-blind, placebo-controlled crossover study demonstrated that caffeine’s cognitive effects in trained volleyball players were both time-of-day dependent and route-specific. Across tasks, oral capsule delivery (3 mg·kg^−1^) produced the most consistent benefits, particularly in the morning and at midday, whereas caffeine mouth rinsing demonstrated a narrower, context-dependent profile with selective effects at midday. These results supported our hypothesis and were in accordance with contemporary sport-nutrition guidance, indicating that caffeine ergogenicity depended on dose, timing, task demands, and individual state [1,48].

Capsules improved simple reaction time at 08:00 and 12:00 versus placebo, while effects were absent at 18:00. For choice reaction time, a benefit emerged only at 12:00, suggesting that added decision demands become facilitative when arousal had naturally rebounded. Regarding executive control, capsule ingestion shortened Stroop interference time at 08:00 and 12:00 and reduced Stroop errors at 08:00, with no between-condition differences in the evening. Caffeine mouth rinsing improved Stroop interference time at 12:00 but did not reliably affect simple reaction time, choice reaction time, or Stroop errors. Taken together, these effects map onto evidence that caffeine robustly enhances vigilance and psychomotor speed, whereas effects on higher-order control are more variable and state-dependent [49,50].

The stronger capsule effects at 08:00 and 12:00 were consistent with circadian and homeostatic processes that degrade alertness and executive function after waking (sleep inertia, lower core body temperature, elevated sleep pressure) and then improved toward the afternoon peak [5,24]. Medium paired-sample effect sizes in the morning and midday (dz ≈ 0.6–0.8 in several contrasts) indicated practically meaningful gains when baseline performance was depressed. By 18:00, the absence of between-condition differences aligned with a ceiling effect, as baseline performance approached daily optima, and headroom for improvement diminished [51]. Within-condition findings (faster evening performance under placebo in some tasks but flattened diurnal variation under capsules) further supported the interpretation that systemic caffeine elevated morning and midday performance toward a more stable plateau rather than enhancing already optimal evening function.

Oral temperature rose from morning to midday (+0.31 °C) but plateaued rather than continuing toward the canonical evening peak, deviating from classic endogenous circadian curves that showed a progressive rise through 18:00–21:00 [52,53]. This pattern likely reflected masking effects (postprandial thermogenesis, prior activity, ambient conditions) superimposed on endogenous rhythmicity, compounded by discrete three-point sampling versus continuous monitoring. Despite this deviation, the midday elevation confirmed time-of-day manipulation validity, and caffeine (3 mg·kg^−1^) did not alter temperature at any time point, indicating ergogenic effects operated independently of acute thermogenic mechanisms at rest [52,53]. Because higher daytime temperature covaried with elevated arousal and improved baseline cognitive readiness, the observed pattern helped to explain why capsule effects were largest at 08:00 and 12:00 (when basal activation and temperature were still rising) and minimal at 18:00, when performance approached daily optima and headroom for improvement narrowed [24,51]. Together with evidence that evening caffeine could phase-delay melatonin without reliably shifting resting temperature, our findings suggested that the cognitive benefits observed here were mediated primarily by adenosine antagonism and state-dependent arousal support rather than by changes in thermoregulation per se [48,54].

Caffeine’s attenuation of diurnal performance variation aligned with prior controlled trials showing preserved function across circadian phases following supplementation [7,25,55]. Souissi et al. [7] demonstrated that 6 mg·kg^−1^ capsules narrowed morning-evening reaction time gaps by elevating suboptimal morning performance toward afternoon baselines. Our 3 mg·kg^−1^ dose produced comparable flattening for SRT (no within-capsule time differences) while preserving some diurnal variation for CRT and Stroop, likely reflecting lower dose magnitude and task-specific sensitivity. This dose-dependent partial compression of circadian amplitude offered a practical advantage: athletes can mitigate early-morning deficits without eliminating physiological rhythmicity that may serve adaptive functions.

Systemic caffeine antagonizes adenosine A_1_ and A_2_A receptors, disinhibiting dopaminergic and noradrenergic signaling that supports attention, response speed, and elements of executive control [4,56]. These canonical central actions plausibly underwrote the capsule’s breadth of effects across simple reaction time and Stroop outcomes [48]. By contrast, caffeine mouth rinsing may have exerted predominantly non-systemic, transient effects via orosensory pathways (oral chemoreceptor activation) and expectancy, though plasma caffeine measurement would be required to confirm negligible systemic absorption following expectoration. Neuroimaging with oral chemosensory stimulation showed rapid central responses (e.g., premotor, insular, orbitofrontal regions), yet these were typically transient and smaller in magnitude than systemic pharmacology [57]. Consistent with this, controlled trials and reviews reported mixed or small performance benefits of caffeine mouth rinsing, with heterogeneity across tasks and protocols [13,15,16].

Our lack of evening between-condition differences was consistent with the findings of Bougrine et al. [23], who reported that caffeine enhanced short-term maximal performance in the morning but not in the evening and that evening dosing increased side effects. Moreover, Bougrine et al. [22] reported dose-dependent benefits on cognitive measures in low caffeine-consuming female athletes, reinforcing that dose and sensitivity shape cognitive outcomes and likely interact with time of day. These findings, together with the present data, supported prioritizing morning and midday dosing and cautious evening use, especially when sleep and recovery are paramount.

Volleyball imposes acute perceptual-cognitive demands under severe temporal pressure. The present time-specific improvements (simple reaction time and Stroop benefits with capsules in the morning and midday, and a selective midday effect of rinsing on interference time) fit with the sport’s neurocognitive requirements and suggested that arousal-supporting interventions offered the greatest return when natural alertness was low or stabilizing (mid-morning to midday) rather than when it was already high (evening) [11]. Even when acute evening performance gains were modest, evening caffeine could still impair sleep (longer latency, lighter sleep, altered architecture) with consequences for recovery and next-day readiness [58,59]. Given the lack of evening between-condition effects here and Bougrine’s report of increased side effects in the evening, routine evening use was difficult to justify unless individualized testing demonstrated net benefit [23].

Interindividual differences in genotype (e.g., CYP1A2, ADORA2A), habitual intake, chronotype, and prior sleep moderated both the magnitude and direction of caffeine’s effects [1,2]. In practice, this supported personalized titration of dose and timing and state-aware use (e.g., larger gains after curtailed sleep; minimal gains when well-rested in the evening). In line with this, dose–response evidence from team-sport athletes indicated that moderate doses can optimize cognitive and physical outputs while limiting adverse events [23,25].

Headache was the sole significantly elevated adverse event (25% at midday post-capsule vs. 0% placebo), with incidence comparable to adult trials at equivalent doses [1,25]. Notably, evening capsule administration (18:00) produced no sleep complaints at 24 h follow-up, contrasting with adult reports of dose-dependent insomnia risk [58,59]. This discrepancy may have reflected adolescents’ faster caffeine metabolism or the 6 h interval between dosing and typical bedtime (~00:00), exceeding two elimination half-lives. Rinse-based delivery produced fewer systemic-type complaints and intermediate perceived improvement, consistent with its likely non-systemic, transient action. In practice, these data supported morning and midday capsule use when cognitive gains were desired, while suggesting caution with evening dosing (sleep risk) and the consideration of mouth rinsing when minimizing systemic exposure is a priority.

For morning training or competition, capsules taken approximately 30–60 min pre-activity appeared most effective for mitigating slower psychomotor speed and weaker executive control [1,60]. For midday sessions, capsules remained effective, and mouth rinsing could be considered when ingestion is impractical (e.g., gastrointestinal sensitivity) or when minimal systemic exposure was desired. For evening activity, any marginal benefit must be weighed against sleep costs [58,59] and against the higher incidence of side effects reported with evening dosing [25]. Consequently, although caffeine can enhance performance at different times of day, its administration must consider both timing relative to circadian rhythms and route of delivery to maximize benefits while minimizing negative consequences [54]. It is worth noting that caffeine should be contextualized as a tactical adjunct rather than a foundational intervention. Optimal sleep quantity and quality (7–9 h nightly in adolescents) exerted substantially greater effects on cognitive and physical performance than any acute ergogenic aid [58,59]. Habitual sleep restriction magnified caffeine’s benefits by deepening morning performance troughs, yet this practice incurred cumulative neurobehavioral costs that episodic supplementation could not remediate. Athletes and practitioners must prioritize sleep hygiene over pharmacological countermeasures, reserving caffeine for scenarios where circadian misalignment or competition scheduling necessitates acute performance support.

### 4.1. Strengths and Limitations

Strengths included the double-blind crossover design, direct comparison of ingestion versus rinsing, and triangulation across multiple cognitive endpoints. Nonetheless, several limitations must be acknowledged. The protocol was not prospectively registered in a clinical trials database, although preregistration is increasingly encouraged in sport science research. Future studies will adopt preregistration via open science platforms to enhance transparency, reproducibility, and analytical accountability. The inclusion of a male adolescent cohort, the use of a fixed dose and timing schedule, the potential taste-cue unblinding during the rinse condition, and the absence of mechanistic biomarkers (plasma caffeine, electroencephalography, core temperature, and objective sleep) limited generalizability and restricted the ability to clarify underlying pathways and state-dependent moderation [15]. Expectancy effects were also uncontrolled. Although the placebo rinse contained quinine to mimic caffeine bitterness, residual taste differences or participant beliefs regarding condition allocation may have influenced outcomes, particularly for the mouth rinse, where orosensory cues inherently compromise blinding. Balanced placebo designs (crossing substance × belief) would isolate pharmacological from psychological effects [1]. The variability in caffeine tolerance and sensitivity due to habitual consumption and genetics were not assessed, further limiting generalizability [61,62]. Individual differences in task engagement and motivational state during cognitive testing were uncontrolled, potentially introducing variance unrelated to circadian or pharmacological factors. Subjective effort ratings or psychophysiological engagement indices (e.g., pupillometry, cardiovascular reactivity) would strengthen future protocols by isolating volitional from pharmacological performance determinants [1,2]. In addition, side effects such as nervousness, anxiety, and sleep perturbation may confound cognitive outcomes and affect validity [58,61].

### 4.2. Future Directions

Future studies should map dose–response and time-course profiles for capsules and rinses by time of day, test combination countermeasures (bright light, brief naps, thermally optimized warm-ups) for additivity in early starts, incorporate chronotype, genotype, and prior-night sleep to derive personalized rules, and embed protocols in match-like environments while co-monitoring sleep and next-day readiness [1,2].

## 5. Conclusions

Isodosed-caffeine ingestion (3 mg·kg^−1^) and caffeine mouth rinsing both improved cognitive performance in well-trained adolescent volleyball players, with effects varying across the day. Caffeine capsules enhanced reaction speed (10–20 ms reductions) and executive control (20–30% interference time decreases), particularly in the morning and at midday. These magnitudes approximate the temporal advantage associated with one additional year of competitive training and may meaningfully influence defensive reaction success during rapid rally exchanges. Mouth rinsing produced smaller, task-specific improvements around midday. Side effects were mild and infrequent, with occasional headaches after capsule ingestion and none after mouth rinsing. Practically, caffeine capsules may help athletes enhance focus and decision-making during periods of lower alertness, whereas mouth rinsing provides a simple, ingestion-free alternative.

## Figures and Tables

**Table 1:** Oral temperature (°C) across time-of-day and supplementation conditions (N = 24).

Time of Day	Placebo	CAFcap	CAFrinse
**8:00**	36.90 ± 0.37	36.95 ± 0.31	36.93 ± 0.35
**12:00**	37.17 ± 0.37 *#	37.20 ± 0.21 *#	37.34 ± 0.24 *#
**18:00**	36.93 ± 0.29	36.95 ± 0.26	37.02 ± 0.33

Values are mean ± SD. CAFcap—caffeine capsule (3 mg·kg^−1^); CAFrinse—caffeine mouth rinse (3 mg·kg^−1^). Measured sublingually (Omron digital thermometer; ±0.05 °C accuracy) with ≥3 min probe retention. * *p* < 0.001 versus 18:00; # *p* < 0.001 versus 08:00 (within-condition comparisons). No between-condition differences at any time point (all *p* ≥ 0.570). Two-way repeated-measures ANOVA: time effect F(2, 46) = 20.90, *p* < 0.0001, ηp^2^ = 0.476; condition effect F(2, 46) = 2.07, *p* = 0.138, ηp^2^ = 0.083; interaction F(4, 92) = 0.43, *p* = 0.784, ηp^2^ = 0.018.

**Table 2:** Cognitive performance outcomes across time-of-day and supplementation conditions (N = 24).

Variable	Time	Placebo	CAFcap	CAFrinse
**SRT (ms)**	8:00	282.70 ± 49.10 *	271.60 ± 43.90 *†	280.70 ± 46.90 *‡
	12:00	279.30 ± 45.50 *#	268.80 ± 42.80 #†§	279.40 ± 43.20 #†‡
	18:00	264.80 ± 39.20	263.80 ± 39.90	263.00 ± 39.70
**CRT (ms)**	8:00	374.00 ± 44.00 *	363.70 ± 43.90 *	371.80 ± 45.60 *
	12:00	360.40 ± 33.60 *#	340.40 ± 29.10 *#†	359.70 ± 41.30 #‡
	18:00	349.50 ± 35.00	351.80 ± 36.00	354.30 ± 32.90
**Stroop Time (s)**	8:00	0.70 ± 0.20 *	0.50 ± 0.20 *†	0.60 ± 0.20 *
	12:00	0.80 ± 0.30 *#	0.50 ± 0.10 *#†§	0.60 ± 0.20 *#‡
	18:00	0.60 ± 0.20	0.60 ± 0.30	0.60 ± 0.20
**Stroop Errors (au)**	8:00	2.30 ± 1.80 *	1.50 ± 1.40 *†	1.80 ± 1.50 **
	12:00	2.00 ± 1.50 *#	1.60 ± 1.40 #††	1.50 ± 1.10 ##
	18:00	1.70 ± 1.70	1.70 ± 1.20	1.70 ± 1.60

Values are mean ± SD. CAFcap—caffeine capsule (3 mg·kg^−1^); CAFrinse—caffeine mouth rinse (3 mg·kg^−1^); SRT—simple reaction time; CRT—choice reaction time; au—arbitrary units. Within-condition: * *p* < 0.001, ** *p* < 0.050 versus 18:00; # *p* < 0.001, ## *p* < 0.050 versus 08:00. Between-condition at same timepoint: † *p* < 0.050, †† *p* < 0.010 versus Placebo; ‡ *p* < 0.050, versus CAFcap; § *p* < 0.050 versus CAFrinse. Statistical details in Section 3.2, Section 3.3, Section 3.4 and Section 3.5.

**Table 3:** Adverse events by time of day and supplementation condition (N = 24).

Symptom	8:00			12:00			18:00		
	Placebo	CAFcap	CAFrinse	Placebo	CAFcap	CAFrinse	Placebo	CAFcap	CAFrinse
Muscle soreness	0.00	0.00	0.00	0.00	0.00	0.00	0.00	0.00	0.00
Increased urinary output	0.00	4.20	0.00	0.00	0.00	0.00	0.00	0.00	0.00
Tachycardia	0.00	0.00	0.00	0.00	0.00	0.00	0.00	0.00	0.00
Anxiety or nervousness	0.00	0.00	0.00	0.00	0.00	0.00	0.00	0.00	0.00
Headache §	4.20	16.70	0.00	0.00	25.00	4.20	0.00	0.00	0.00
Gastrointestinal problems	0.00	0.00	0.00	0.00	0.00	0.00	0.00	0.00	0.00
Insomnia	0.00	0.00	0.00	0.00	0.00	0.00	0.00	0.00	0.00
Increased activity	0.00	8.30	0.00	0.00	0.00	0.00	0.00	0.00	0.00
Perceived performance improvement §	12.50	33.30	20.80	12.50	50.00 *	33.30	12.50	37.50	37.50

Note. Values are percentages of participants reporting each symptom. CAFcap—caffeine capsule (3 mg·kg^−1^); CAFrinse—caffeine mouth rinse (3 mg·kg^−1^). * Significant within-time-of-day difference for CAFcap versus Placebo (*p* < 0.050). § Significant overall difference across conditions within at least one time point (Cochran’s Q, *p* < 0.050).

## Data Availability

The original contributions presented in this study are included in the article. Further inquiries can be directed to the corresponding authors.
